# The PD COMM trial: a protocol for the process evaluation of a randomised trial assessing the effectiveness of two types of SLT for people with Parkinson’s disease

**DOI:** 10.1186/s13063-017-2130-1

**Published:** 2017-08-29

**Authors:** Patricia Masterson-Algar, Christopher R. Burton, Marian C. Brady, Avril Nicoll, Carl E. Clarke, Caroline Rick, Max Hughes, Pui Au, Christina H. Smith, Catherine M. Sackley

**Affiliations:** 1 Bangor Institute for Health & Medical Research, School of Healthcare Sciences, Bangor University, Ffriddoedd Road, Bangor, UK; 2 Nursing, Midwifery and Allied Health Professions Research Unit, Glasgow Caledonian University, Glasgow, UK; 3 Institute for Applied Health Research, University of Birmingham, Vincent Drive, Birmingham, UK; 4 Department of Neurology, Sandwell and West Birmingham Hospitals NHS Trust, Birmingham, UK; 5 Birmingham Clinical Trials Unit (BCTU), University of Birmingham, Edgbaston, Birmingham, UK; 6 Faculty of Brain Sciences, University College London, London, UK; 7 Faculty of Life Science and Medicine, King’s College London, London, UK

**Keywords:** Process evaluation, Complex interventions, Speech and language therapy, Lee Silverman Voice Treatment, Fidelity

## Abstract

**Background:**

The PD COMM trial is a phase III multi-centre randomised controlled trial whose aim is to evaluate the effectiveness and cost-effectiveness of two approaches to speech and language therapy (SLT) compared with no SLT intervention (control) for people with Parkinson’s disease who have self-reported or carer-reported problems with their speech or voice. Our protocol describes the process evaluation embedded within the outcome evaluation whose aim is to evaluate what happened at the time of the PD COMM intervention implementation and to provide findings that will assist in the interpretation of the PD COMM trial results. Furthermore, the aim of the PD COMM process evaluation is to investigate intervention complexity within a theoretical model of how the trialled interventions might work best and why.

**Methods/design:**

Drawing from the Normalization Process Theory and frameworks for implementation fidelity, a mixed method design will be used to address process evaluation research questions. Therapists’ and participants’ perceptions and experiences will be investigated via in-depth interviews. Critical incident reports, baseline survey data from therapists, treatment record forms and home practice diaries also will be collected at relevant time points throughout the running of the PD COMM trial. Process evaluation data will be analysed independently of the outcome evaluation before the two sets of data are then combined.

**Discussion:**

To date, there are a limited number of published process evaluation protocols, and few are linked to trials investigating rehabilitation therapies. Providing a strong theoretical framework underpinning design choices and being tailored to meet the complex characteristics of the trialled interventions, our process evaluation has the potential to provide valuable insight into which components of the interventions being delivered in PD COMM worked best (and what did not), how they worked well and why.

**Trial registration:**

ISRCTN Registry, ISRCTN12421382. Registered on 18 April 2016.

**Electronic supplementary material:**

The online version of this article (doi:10.1186/s13063-017-2130-1) contains supplementary material, which is available to authorized users.

## Background

### PD COMM trial

Parkinson’s disease (PD) is one of the most common serious movement disorders in the world [1, 2], affecting approximately 120,000 people in the United Kingdom alone [3]. Speech impairments, which affect a large proportion of the PD population, have been recognised to reduce these individuals’ quality of life [4, 5]. The PD COMM trial is a phase III, multicentre, three-arm, unblinded randomised controlled trial (RCT) whose aim is to evaluate the effectiveness, cost-effectiveness and implementation process of two types of speech and language therapy (SLT) compared with no treatment (control) for people with PD who have self-reported problems with their speech or voice. It will also compare the two types of SLT (Lee Silverman Voice Treatments [LSVT] versus NHS standard SLT). Results derived from a pilot trial (PD COMM Pilot) informed the design of this RCT (Sackley et al: Lee Silverman voice treatment versus standard speech and language therapy versus control in Parkinson’s disease: a pilot randomised controlled trial, submitted); a full description of the pilot trial is provided elsewhere [6].

Although a Cochrane review was unable to support the efficacy of any form of SLT over another, owing to small participant numbers and methodological flaws in trials [7], LSVT is regarded as the most efficacious treatment for communication impairments in patients with PD [8]. Its aim is to improve phonation and vocal loudness through better vocal fold adduction [9]. As recommended by the originators, LSVT will be administered in four 50-minute sessions per week for 4 weeks. Each session will consist of 25 minutes of repeated and intensive maximum-effort drills and 25 minutes of high-effort speech production tasks [9]. Participants will be asked to complete up to 10 minutes of home-based practice/tasks on treatment days and up to 30 minutes on non-treatment days [10]. The second trial intervention, NHS standard SLT, will be individualised to suit the needs of each participant and will reflect local practice. Thus, its dose and frequency will be more variable, but it will likely consist of one session per week for 6–8 weeks of varying content which may include behavioural compensatory strategies and augmentative and alternative communication strategies to improve functional communication and participation [11].

### Investigating the complexity of PD COMM interventions via process evaluation

This paper describes the protocol for the process evaluation that is being conducted alongside the outcome evaluation as part of the PD COMM trial. In order to assess fidelity, this process evaluation will be focused on investigating the quality of implementation of the PD COMM interventions as well as adherence to the outcome evaluation protocol. As the Medical Research Council guidance [12, 13] states, process evaluations serve a vital role because they are focused on identifying the underpinning characteristics of intervention components and how these impact delivery of the intervention to a set standard. They also provide detailed insight into the experiences of those who engage with the complex intervention [14, 15]. Critically, by carrying out a process evaluation, it is possible to identify if observed impacts are due solely to the trial intervention or are a result of a number of external and internal variables that are closely linked to the environment and the context in which the interventions take place [15–18]. Finally, complex trials such as PD COMM which include a process evaluation will produce higher-quality results that not only can improve theory-informed rehabilitation interventions but also help increase their potential generalizability and optimisation in routine practice [19, 20].

In order to identify the causal processes taking place during the PD COMM trial, this process evaluation has used the Normalization Process Theory (NPT), which is highly attuned to the challenges of complex interventions [21]. This theory provides a robust analytical framework to examine how speech and language therapists as well as trial participants embed and integrate (sustain) PD COMM interventions into their routine practice and daily life, respectively [22]. This framework [23] explains how this is accomplished through four generative mechanisms (coherence, cognitive participation, collective action and reflexive monitoring). By using the NPT framework, we consider that we can explore, describe and understand the processes that are taking place and the interactions and gaps between the PD COMM interventions, the changing context, speech and language therapists and their practice.

### Aims and objectives

The overall aim of this process evaluation is to evaluate and understand what worked and why at the time of the PD COMM intervention implementation and to provide findings that will assist in the interpretation of the PD COMM trial results. Specific objectives are as follows:

1. Establish a programme theory that explains how the PD COMM complex interventions are expected to work to generate outcomes amongst a complex patient group. This programme theory will consist of a set of tangible assumptions that reflect the multi-component nature of the PD COMM interventions. It will further be used to guide the analysis and synthesis of data.
2. Investigate the implementation of trial interventions from the perspective of therapists. This will include attention to previous experience, level of acceptance of the interventions, and therapists’ expectations.
3. Investigate study participants’ experiences and views about their involvement in the PD COMM study, identifying factors shaping and differentiating life with PD, participation, motivation, and (where relevant) adherence to therapy for those in the treatment and control groups.

## Methods/design

The process evaluation will be conducted as an integral part of the PD COMM trial and will use NPT to examine normalisation from the perspectives of both staff delivering the intervention and patient participants who need to embed the trial interventions into their daily routines. A research officer will lead the work as part of a small sub-team of process evaluation researchers who will monitor its progress and contribute to the ongoing data analysis. Throughout the PD COMM trial, the process evaluation team will also be in regular contact with LSVT developers in order to discuss adherence to LSVT protocols and quality of implementation. A mixed method design will be used to address research objectives. This will involve the integration of both qualitative and quantitative data in the research process.

### Data collection

We will employ a number of approaches to data collection during the PD COMM process evaluation in order to answer our research questions (Tables 1 and 2).

**Table 1:** Data collection methods and time points

Data collection method	Time points
Therapist questionnaire (TQ)	Prior to start After therapists have treated their last PD COMM participant
Observations of therapists’ LSVT training and additional workshops organized by the trial researchers	Prior to start Yearly workshops
Semi-structured in-depth interviews	
• With participants	After 3-month assessment
• With speech and language therapists and SLT assistants	Two time points: midway and at the end of therapists’ involvement in the trial
Critical incident reports (CIRs)	Throughout therapists’ involvement in the trial
SLT treatment record form (SLT TRF)	Every therapy session
Standard NHS SLT home-based therapy (HBT) diary	Completed when home-based therapy is prescribed by speech and language therapists
LSVT home-based therapy (HBT) diary	Completed once for each of the 4 weeks of LSVT

*LSVT* Lee Silverman Voice Treatments, *SLT* Speech and language therapy

**Table 2:** Data sources used to address PD COMM process evaluation objectives and analytical questions

Objectives	Analytical questions	Data sources
Establish a programme theory that explains how PD COMM interventions are expected to work	What are the components of the PD COMM interventions? Which are core? Which are peripheral? What are the interactions between the components of the PD COMM interventions?	OBS, TQ, therapists’ interviews, TRF, HBT diaries
Investigate the implementation of trial interventions from the perspective of therapists	What were therapists’ opinions of the PD COMM interventions?	Therapists’ interviews, CIRs, OBS
	What experience of delivering the PD COMM interventions did the therapists have prior to the start of the trial?	TQ, therapists’ interviews
	What were therapists’ motivations for joining the trial?	TQ, therapists’ interviews
	To what extent did the training help therapists ‘feel ready’?	OBS, therapists’ interviews
	How did ‘learning time’ impact the way therapists delivered the PD COMM interventions?	TQ, therapists’ interviews, TRF
	To what extent did therapists feel supported in their role as research therapists?	TQ, therapists’ interviews, CIRs
	What challenges did therapists face? How did they manage them?	OBS, therapists’ interviews, CIRs
	How did therapists feel about the way they recorded content and results from therapy sessions?	Therapists’ interviews, TRF
	How did therapists work on building rapport with participants?	Therapists’ interviews, TRF, CIRs
	How did the PD COMM interventions compare with the therapists’ routine way of practicing?	TQ, therapists’ interviews, CIRs
	What are the therapists’ views regarding the resources allocated to the trial?	TQ, therapists’ interviews
	How have therapy outcomes impacted the therapists’ level of motivation/engagement?	Therapists’ interviews, CIRs
	To what extent did therapists tailor the PD COMM interventions to participants’ needs?	TQ, therapists’ interviews, TRF, CIRs, HBT diaries
	How did therapists feel about their research role versus their client-centred approach to practice?	Therapists’ interviews, CIRs
	To what extent did therapists follow the trial intervention protocol?	TQ, therapists’ interviews, TRF
	To what extend did therapists feel supported by their environment?	TQ, therapists’ interviews, CIRs
Investigate participants’ experiences and views about their involvement in the PD COMM trial	To what extent did participants engage in the trial?	Participants’ interviews, HBT diaries
	What did participants think of their involvement in the trial?	Participants’ interviews
	How did the therapy sessions impact their everyday routine?	Participants’ interviews, HBT diaries
	What were participants’ motivations for joining the trial?	Participants’ interviews
	Can participants identify positive/negative impacts of their involvement in the trial?	Participants’ interviews

*Abbreviations: CIR* Critical incident report, *HBT* Home-based therapy, *LSVT* Lee Silverman Voice Treatments, *OBS* Observations of Lee Silverman Voice Treatments training and trial workshops, *SLT* Speech and language therapy, *TQ* Therapist questionnaire, *TRF* Treatment record form

#### Therapist questionnaire

All speech and language therapists and SLT assistants involved in the trial will be asked to complete an online questionnaire at two time points: prior to the start of intervention delivery and after they have treated their last PD COMM participant. Guided by the NPT constructs [23, 24], this questionnaire (*see* Additional file 1) includes three sections with a series of Likert scale questions which will explore the following:

1. Therapists’ role within their service, previous experience as speech and language therapists and SLT assistants delivering LSVT and NHS standard therapy, and information on relevant training
2. Therapists’ understanding of LSVT and NHS standard therapy interventions and their familiarity in their implementation, both at a personal level and in terms of the working context; also included are therapists’ expectations in regard to their ability to carry out their research role, being aware of potential deviations from their usual ways of working
3. Therapists’ opinions on how they feel their skills have developed in regard to delivering LSVT and NHS standard therapy

In order to measure therapists’ self-belief, the questionnaire includes the Generalized Self-Efficacy Scale [25] in one of its sections. The last part of the therapist questionnaire is an LSVT practice checklist (copyright 2014 by LSVT Global, Inc., Tucson, AZ, USA) which has been created by the intervention developers and is divided into eight sections outlining the LSVT intervention. This skills checklist will provide valuable insight into therapists’ level of confidence in their ability to deliver all components of LSVT.

#### Observations of therapists’ training and trial workshops

Observations by the process evaluation research officer will be made during the LSVT training and certification courses, which will be offered to both new and previously certified clinicians prior to their involvement in the PD COMM trial. The views of LSVT intervention developers providing the training will also be recorded on the day of the training. Furthermore, all other workshops organized by the trial researchers with network therapists will be observed. The purpose of these workshops will be to help build a network of research therapists and generate enthusiasm around engagement with PD COMM. Therapists, research nurses and researchers attending the workshops will be able to voice their concerns and discuss potential strategies to address barriers to implementation. It is anticipated that one event per year will be scheduled throughout the running of the trial. These observations will provide substantial data on the therapists’ level of involvement and engagement in the trial. During the observations, the research officer will make field notes guided by Spradley’s [26] work, which identifies nine dimensions of descriptive observation and encourages the researcher to focus on things that are not necessarily visible. Particular attention will be given to issues centred on goal setting, skill acquisition and competence.

#### Semi-structured in-depth interviews

##### Study participants

Face-to-face qualitative interviews will be conducted with a purposive sample of 20 participants in each of the 3 trial arms. Sampling will ensure engagement of trial participants with different ages and PD severities (Hoehn & Yahr stage) as well as the presence of a family carer. Participants will be invited to take part in the interview at a time and place that suits them. Interviews should last no longer than 30 minutes. All participants will be given an information sheet and will be asked to sign a consent form prior to the interview.

Interviews will take place after the three month assessment, and will draw on an interview spine underpinned by both the NPT analytical framework [23] and the PD COMM intervention programme theory (*see* Additional file 2). By being focused on the work associated with assimilating new interventions into pre-existing norms and routines, the use of this theoretical perspective will enable the differentiation of managing life with PD in general and speech and therapy interventions in particular.

##### Study therapists

Semi-structured in-depth interviews will be conducted with speech and language therapists and SLT assistants delivering the two active treatment arms. The programme theory will guide questions exploring issues linked to the implementation of this complex intervention from the therapists’ perspectives. The structure and content of these interviews will similarly reflect the four mechanisms described in the NPT analytical framework [23] in order to understand the work of therapists in delivering and embedding the intervention in their routine practice. All participants will be asked to give consent to the recording prior to the start of the interview, which will be carried out over the phone at a convenient date and time. Interviews are estimated to last a maximum of 1 h. All interviews will be digitally recorded and fully transcribed and then checked by the interviewer, keeping all names of participants anonymous. As a recommended quality assurance measure, transcripts will be made available to all participants (member checking) as a way to ensure the validity and accuracy of findings [27]. Therapists will also be given the opportunity to provide subsequent reflections if they consider them necessary.

Interviews will be carried out at two time points: approximately midway through therapists’ involvement in the trial and at the end of their involvement, once they have treated their last PD COMM participant. This will be essential in order to discern the impact that learning over time might have on therapists’ role in delivering the PD COMM interventions. Prior to the interview, written information will be sent to all speech and language therapists via an email explaining the aims of the process evaluation and the reasons underlying the importance of collecting data via individual interviews. Therapists will be encouraged to talk openly and discuss failures and problems as well as successes. If they wish, they can also talk more abstractly about what might have occurred had things been different. For a copy of the interview schedule, *see* Additional file 3.

#### Critical incident reports

All therapists will be asked to record key reflections using a critical incident technique [28]. Data will be collected in the form of critical incident reports (CIRs) and will follow Gibbs’s [29] reflective cycle. (A copy of the CIR form is provided in Additional file 4.) Guiding questions in the form are designed to encourage therapists to examine their own assumptions and beliefs as well as the reasoning and values underpinning their practice [30]. The aim is to further investigate the sense that therapists make of particularly challenging or memorable situations that take place during their involvement in the PD COMM trial. CIR data will reveal therapists’ ‘theories in use’ rather than any espoused practice to conform to researchers’ expectations of their work within the trial. Speech and language therapists and SLT assistants will be briefed regarding the purpose and content of CIRs and will be asked to submit their completed forms by post or by email throughout their involvement in the trial. Reminder emails will be sent to the therapists in order to encourage completion.

#### SLT treatment record form

All speech and language therapists and SLT assistants involved in the PD COMM trial will be asked to complete an SLT treatment record form (TRF). This record represents a data source from the outcome evaluation which will be further used for the purpose of the process evaluation. In this SLT TRF, therapists working in each of the study sites will record the number of minutes they dedicate to various aspects of the PD COMM interventions at each therapy session.

These SLT TRFs will be used to monitor participant adherence (e.g., missed or cancelled appointments) and therapist adherence to the protocols for these programmes. In addition, for the standard SLT intervention, the forms will be used to further explore what standard SLT delivered within the NHS entails.

#### Home-based therapy diaries

Participants in each of the intervention arms will be asked to complete home-based therapy (HBT) diaries. Those participants receiving LSVT will be asked to complete an LSVT HBT diary for each week of treatment. Each diary is used to record information for 7 days of home LSVT practice exercises. Those participants receiving standard NHS SLT will be asked to complete a diary as and when they are prescribed home-based exercises by their therapist. These HBT diaries will measure the amount of intervention practice that participants complete outside formal intervention delivery sessions.

### Data analysis

The analysis and synthesis of data will be driven by the programme theory that explains the PD COMM interventions and their impact over time. Both qualitative and quantitative methods will be used to describe the multiple, multi-level, interacting components within and across both PD COMM intervention arms. Although components of the intervention are well specified in LSVT, in NHS standard SLT they are not, as reported in the PD COMM Pilot [6].

#### Quantitative data

Both intervention adherence and intervention logic require a traditional approach to data analysis, being focused on within- and between-group comparisons. The heterogeneity and lack of a benchmark for standard NHS SLT places limits on the extent to which adherence and quality of implementation (fidelity) can be monitored. However, data derived from the SLT TRF and the HBT diaries will allow the process evaluation team to describe the following statistically within and between each intervention arm:

- What NHS standard SLT entails
- How therapists structured the intervention (including who delivered it and where as well as how time was reportedly allocated to different aspects specified by the research team)
- How regularly participants attended scheduled appointments
- How closely participants reported adhering to the type and dosage of home practice activities agreed between them and their therapist, and whether they attended adjuncts to therapy such as group activities

In addition, the process evaluation team will aim to describe statistically how the structure of intervention content (in terms of the distribution of time spent on aspects specified by the research team) shifted over the course of intervention for participants. This will be for each intervention arm as a whole because it is not considered likely that either participating therapists or centres will have sufficient numbers of participants to enable meaningful within-group comparisons.

The team will categorise, describe and identify trends in the use of components of the PD COMM interventions by systematically extracting data from the SLT TRF, HBT diaries and the therapy notes, mapping the data to the Template for Intervention Description and Replication framework [31] and exploring these data statistically. To ensure the findings have face validity and continue to be grounded in practice, constant feedback from a network of participating PD COMM therapists will be sought.

Descriptive statistics will be analysed with the data derived from the therapist questionnaire. This will indicate the frequency of responses to items and will show where therapists have shown a more positive or negative responses.

#### Qualitative data

All interviews will be recorded and transcribed verbatim after anonymization. The Atlas.ti software package (ATLAS.ti Scientific Software Development GmbH, Berlin, Germany) will be used to facilitate analysis.

Intervention fidelity and complexity are associated with different analytical approaches, which may integrate different types of data from different sources. These analyses are associated with the development of a theoretical understanding of issues pertinent to how the interventions operate in the context of the clinical trial. All interview data, CIRs and notes from observations will be coded using a theoretical coding framework underpinned by the PD COMM intervention programme theory and NPT constructs [21]. The analysis will follow the phases of thematic analysis described by Braun and Clarke [32]. First, two of the authors (PMA and CRB) will re-read all transcripts and notes to gain familiarity with the data. The researchers will then code all data informed by the coding framework and will assign relevant data extracts to each code. The distribution of codes will be recorded, and new codes will be created for data falling outside the coding framework to avoid missing important concepts. Themes will then be identified as meaningful patterns across coded data. Themes will then be reviewed and agreed by all authors. These qualitative data analyses will be conducted within source prior to synthesis across data sources.

#### Synthesis

To allow for synthesis, the initial framework of thematic categories generated from the thematic analysis will be applied to all data as part of the analytical approach. The analytical task will be to describe the acceptability of participants’ engagement with the study interventions, differentiation of management of life with PD in general, and speech and therapy interventions in particular. The quantitative data on intervention delivery and adherence, together with qualitative insights into participants’ engagement in the trial, quality of implementation and complexity issues, will be triangulated to develop an explanatory account of the outcome evaluation findings. Data analyses will be ongoing, and process data will be analysed independently of the outcome evaluation data before the two sets of data are then combined.

### Governance

The PD COMM trial outcome and process evaluation will be effectively integrated to avoid, for example, duplication of effort. However, some of the process evaluation data collection will be carried out at ‘arm’s length’ in order to reduce the burden on participants. Researchers at Glasgow Caledonian University will collect and analyse intervention component data and adherence to the prescribed interventions (SLT TRF, HBT diaries and therapy notes). Researchers from Bangor University will collect all qualitative data, including the therapist questionnaire, in order to investigate all aspects of quality of implementation of the PD COMM complex interventions. The process evaluation team will produce 6-monthly reports on progress to the trial management team.

### Ethics and consent

Ethical approval for this process evaluation has been obtained from the Bangor University Ethics Committee. Information about the process evaluation has been included in the PD COMM trial protocol, information sheets and consent forms. Participants will have the option to consent to be contacted regarding taking part in the process evaluation at the time of consenting to take part in the PD COMM trial. Ethical approval for the PD COMM trial was given on 17 December 2015 (REC 15/WM/0443).

## Discussion

Investigating fidelity via high-quality process evaluations can increase researchers’ understanding of why a complex intervention works or fails. To date, there are a limited number of published process evaluation protocols, and the number is even lower when linked to trials investigating rehabilitation therapies. Regardless of this, there has been a significant increase in the funding allocated to carry out process evaluations. There is no single way to design and carry out a process evaluation [17, 33]; this is particularly true when dealing with complex interventions such as the ones investigated in the PD COMM trial. Hence, the research team faced having to make choices about what aspects of the interventions and their delivery to focus on, as well as what methods to select in order to address these whilst avoiding the collection of unnecessary data at a high cost [34]. By having a strong theoretical framework underpinning our design choices and by tailoring the evaluation to meet the intrinsic characteristics of this trial, this process evaluation can potentially lead to a good understanding of not only how the PD COMM interventions worked best and why but also for whom and where. The aims of PD COMM are to achieve this and bring clarity to how therapists’ learning and tailoring throughout the implementation of a complex intervention can impact outcomes. Rehabilitation interventions such as PD COMM interventions are likely to be tailored as therapists become more experienced and ‘learn’ how to best target participants’ needs whilst staying true to the trial’s intervention protocol. In today’s healthcare research, where client-centredness plays a major role, there is an increased awareness of the need to tailor interventions to patients’ needs and cultural backgrounds [35]. This process evaluation will have a role in monitoring how this is done whilst exploring individual contexts, and this may help to explain variations in the effectiveness of the intervention.

## Additional files

Additional file 1: PD COMM therapist questionnaire. (PDF 787 kb)

Additional file 2: PD COMM process evaluation participant interview schedule. (PDF 354 kb)

Additional file 3: PD COMM process evaluation therapist interview schedule. (PDF 369 kb)

Additional file 4: PD COMM process evaluation critical incident report form. (PDF 291 kb)

## Abbreviations

CIR: Critical incident report
HBT: Home-based therapy
LSVT: Lee Silverman Voice Treatments
NPT: Normalization Process Theory
OBS: Observations of Lee Silverman Voice Treatments training and trial workshops
PD: Parkinson’s disease
RCT: Randomised controlled trial
SLT: Speech and language therapy
TQ: Therapist questionnaire
TRF: Treatment record form
