# Supplementary material for: The PD COMM trial: a protocol for the process evaluation of a randomised trial assessing the effectiveness of two types of SLT for people with Parkinson’s disease
Source: Trials. 2017 Aug 29;18:397. doi: 10.1186/s13063-017-2130-1 (PMC5576370; doi:10.1186/s13063-017-2130-1)
Supplement: Supplementary file 1 — PD COMM therapist questionnaire. (PDF 787 kb) [file 13063_2017_2130_MOESM1_ESM.pdf]

Therapist Questionnaire

OVERVIEW

As a speech and language therapist or therapy assistant working on the study you may be responsible for delivering Lee Silverman Voice Treatment (LSVT), NHS standard speech and language therapy or both. In this survey we would like you to provide us with information that will help us build a picture of how you feel about your role and responsibilities within the PD COMM trial. The questions are designed to help us get a better understanding of potential issues around the implementation of a complex intervention such as speech and language therapy for Parkinson's disease.

This questionnaire is divided in 3 sections:

· SECTION A asks some questions about yourself and your role as a speech and language therapist or therapy assistant.

· SECTION B asks questions regarding how you feel about using LSVT or/and “NHS standard therapy” as part of your work.

· SECTION C asks questions about how you feel that you have developed or are developing the particular sets of skills required to deliver LSVT.

Please take time to decide which answer best suits your experience. Thank you very much for your help.

Therapist Questionnaire

SECTION A

About yourself

Contact Information

Name

Email Address

What Trust do you work for?

A1. How many years have you worked for this Trust/Organization *(if your Trust has merged with another or changed its name, please include in your answer the total time you have worked with this Trust and its predecessors)*

- ◯ Less than one year
- ◯ 1-2 years
- ◯ 3-5 years
- ◯ 6-10 years
- ◯ 11-15 years
- ◯ More than 15 years

A2. Are you a:

- ◯ Speech and language therapist (SALT)
- ◯ Speech and language therapy assistant (SALT assistant)

A3. What is your current job title and grade?

A4. How many years have you been practicing as a SALT or SALT assistant?

- Less than one year
- 1-2 years
- 3-5 years
- 6-10 years
- 11-15 years
- More than 15 years

A5. How many years of experience working with PD patients have you got?

- Less than one year
- 1-2 years
- 3-5 years
- 6-10 years
- 11-15 years
- More than 15 years

A6. Do you deliver LSVT as part of your current role in your Trust?

- Yes
- No

A7. Have you received LSVT training?

- No - please go to question A9
- Yes - please go to question A8

A8. If yes, when:

A9. How many years of experience delivering LSVT have you got?

- ◯ I have just completed my training
- ◯ Less than one year
- ◯ 1-2 years
- ◯ 3-5 years
- ◯ 6-10 years
- ◯ 11-15 years
- ◯ More than 15 years

A10. During your career, have you been involved in the treatment of a minimum of three patients using LSVT?

- ◯ Yes
- ◯ No - please contact the Trial Manager for advice and complete the rest of the questionnaire considering your skills and experience of being a speech and language therapist (or therapy assistant) and how you anticipate your work delivering LSVT and NHS standard therapy will be like.

Therapist Questionnaire

SECTION B

As part of your role in the PD COMM study you may be responsible for treating patients using LSVT or NHS standard therapy. The following survey items are designed to explore how familiar each of these interventions feel to you. You will be asked to think about each intervention separately and provide information regarding how familiar its use and its content is to you, how you will be able to use it in your normal work and how you think you will be able to cope with a variety of demands linked to its delivery.

Section B1

In regards to delivering NHS standard therapy

When you use **NHS standard therapy**, how familiar does it feel?

0 - Still feels very new | 5 | 10 - Feels completely familiar

Do you feel **NHS standard therapy** is currently a normal part of your work?

0 - Not at all | 5 - Somewhat | 10 - Completely

Do you feel **NHS standard therapy** will become a normal part of your work?

0 - Not at all | 5 - Somewhat | 10 - Completely

Please take time to decide which answer best suits your experience of using**NHS standard therapy**

	Strongly agree	Agree	Neither agree or disagree	Disagree	Strongly disagree
I believe that treating patients using **NHS standard therapy** is a legitimate part of my role	◯	◯	◯	◯	◯
I can see how **NHS standard therapy** differs from usual ways of working with speech disorders in the context of SLT interventions	◯	◯	◯	◯	◯
SALT and SALT assistants in my Trust have a shared understanding of the purpose of **NHS standard therapy**	◯	◯	◯	◯	◯
I understand how treating patients using **NHS standard therapy** affects the nature of my own work	◯	◯	◯	◯	◯
I can easily integrate **NHS standard therapy** into my existing work	◯	◯	◯	◯	◯
I can see the potential value of **NHS standard therapy** into my existing work	◯	◯	◯	◯	◯
There are key people who drive the use of **NHS standard therapy** forward and get others involved	◯	◯	◯	◯	◯
I am open to working with colleagues in new ways to be able to treat patients using **NHS standard therapy**	◯	◯	◯	◯	◯
I will continue to support **NHS standard therapy**	◯	◯	◯	◯	◯
The use of **NHS standard therapy** to treat patients disrupts working relationships	◯	◯	◯	◯	◯
I have confidence in other people's ability to deliver **NHS standard therapy**	◯	◯	◯	◯	◯

	Strongly agree	Agree	Neither agree or disagree	Disagree	Strongly disagree
Work is assigned to those with the right skills necessary to treat patients using **NHS standard therapy**	◯	◯	◯	◯	◯
Sufficient training is provided to enable staff to deliver **NHS standard therapy**	◯	◯	◯	◯	◯
Sufficient resources are available to support the delivery of **NHS standard therapy**	◯	◯	◯	◯	◯
The management team adequately supports the use of **NHS standard therapy**	◯	◯	◯	◯	◯
I am aware of research about the effects of **NHS standard therapy**	◯	◯	◯	◯	◯

Therapist Questionnaire

Section B2

In regards to delivering LSVT

When you use **LSVT**, how familiar does it feel?

0 - Still feels very new	5	10 - Feels completely familiar

Do you feel **LSVT** is currently a normal part of your work?

0 - Not at all	5 - Somewhat	10 - Completely

Do you feel **LSVT** will become a normal part of your work?

0 - Not at all	5 - Somewhat	10 - Completely

Please take time to decide which answer best suits your experience of using **LSVT**

	Strongly agree	Agree	Neither agree or disagree	Disagree	Strongly disagree
I believe that treating patients using **LSVT** is a legitimate part of my role	○	○	○	○	○
I can see how **LSVT** differs from usual ways of working with speech disorders in the context of SLT interventions	○	○	○	○	○

	Strongly agree	Agree	Neither agree or disagree	Disagree	Strongly disagree
SALT and SALT assistants in my Trust have a shared understanding of the purpose of **LSVT**	○	○	○	○	○
I understand how treating patients using **LSVT** affects the nature of my own work	○	○	○	○	○
I can easily integrate **LSVT** into my existing work	○	○	○	○	○
I can see the potential value of **LSVT** into my existing work	○	○	○	○	○
There are key people who drive the use of **LSVT** forward and get others involved	○	○	○	○	○
I am open to working with colleagues in new ways to be able to treat patients using **LSVT**	○	○	○	○	○
I will continue to support **LSVT**	○	○	○	○	○
The use of **LSVT** to treat patients disrupts working relationships	○	○	○	○	○
I have confidence in other people's ability to deliver **LSVT**	○	○	○	○	○
Work is assigned to those with the right skills necessary to treat patients using **LSVT**	○	○	○	○	○
Sufficient training is provided to enable staff to deliver **LSVT**	○	○	○	○	○
Sufficient resources are available to support the delivery of **LSVT**	○	○	○	○	○
The management team adequately supports the use of **LSVT**	○	○	○	○	○
I am aware of research about the effects of **LSVT**	○	○	○	○	○

Therapist Questionnaire

Section B3

In regards to how you deal with your everyday job as a SALT or SALT assistant

	Not at all true	Hardly true	Moderately true	Exactly true
I can always manage to solve difficult problems if I try hard enough	○	○	○	○
If someone opposes me, I can find the means and ways to get what I want	○	○	○	○
It is easy for me to stick to my aims and accomplish my goals	○	○	○	○
I am confident that I could deal efficiently with unexpected events	○	○	○	○
Thanks to my resourcefulness I know how to handle unforeseen situations	○	○	○	○
I can solve most problems if I invest the necessary effort	○	○	○	○
I can remain calm when facing difficulties because I can rely on my coping abilities	○	○	○	○
When I am confronted with a problem, I can usually think of a solution	○	○	○	○
If I am in trouble, I can usually think of a solution	○	○	○	○
I can usually handle whatever comes my way	○	○	○	○

Therapist Questionnaire

SECTION C

LSVT is a complex intervention and requires speech and language therapists and therapy assistants to develop a series of skills in order to deliver it. In this section we want to find out how you feel your skills have developed in regards to delivering LSVT. Please think about your experience of delivering LSVT and choose the answer that best describes how you feel about the following set of skills

C1. Daily variables

	"I've got it" (Well-developed skill)	***"I am getting it"*** (Emerging skill)	***"I don't get it"*** (Need help!)
Encouraging maximal muscle training with repetitions; 15 repetitions of Ahs, Highs and Lows, 5 repetitions of 10 Functional Phrases, with pauses only for shaping/modelling productions (may encourage more repetitions if time permits)	○	○	○
Reverting to model ("Do what I do") anytime non-target performance observed	○	○	○
Pushing client performance appropriately	○	○	○
Providing verbal feedback to motivate motor performance and sensory awareness	○	○	○
Completing daily core exercises in 25-30 minutes	○	○	○
Focusing on goal of LOUD and GOOD QUALITY phonation	○	○	○
Relating effort to new loudness level	○	○	○
Ensuring client is using or starting with modal pitch of Ahs, Highs and Lows	○	○	○
Asking follow-up questions as a way to monitor (and shape if needed) off the cuff speech	○	○	○

C2. Shaping client behaviour

	"I've got it" (Well-developed skill)	***"I am getting it"*** (Emerging skill)	***"I don't get it"*** (Need help!)
Watching client instead of paper/instrumentation	◯	◯	◯
Modelling with “Do what I do”	◯	◯	◯
Limiting verbal cues to Loud/Effort	◯	◯	◯
Providing visual cues for client needs. Examples: open mouth, head/neck posture, overall posture, breath support, intonation/pitch variation, decreased head, neck, shoulder tension	◯	◯	◯
Using additional therapeutic techniques as needed (e.g. easy onsets, isometric exercises, etc.)	◯	◯	◯
Dropping cues once client demonstrates behaviour	◯	◯	◯
Watching for signs of hyperfunction and shaping accordingly	◯	◯	◯
Targeting loud, good quality voice in all activities	◯	◯	◯

C3. Data tracking

	"I've got it" (Well-developed skill)	***"I am getting it"*** (Emerging skill)	***"I don't get it"*** (Need help!)
Tracking data (SPL, frequency, duration) while providing feedback about performance and without distracting client	○	○	○
Tracking data for off the cuff speech to determine client progress and to assess calibration	○	○	○
Balancing instrumental data with subjective clinical impressions	○	○	○
Measuring mouth to microphone distance throughout each session to ensure distance is consistent within and across sessions	○	○	○
Using data to quantify client progress in documentation	○	○	○

C4. Calibration

	"I've got it" (Well-developed skill)	***"I am getting it"*** (Emerging skill)	***"I don't get it"*** (Need help!)
Calibrating EVERY off the cuff speech act if not target loudness	◯	◯	◯
Reinforcing to client that you are training healthy vocal loudness, within normal limits	◯	◯	◯
Noting self-correction of off the cuff speech as progress in internal cueing	◯	◯	◯
Using recording and playback to further enhance calibration	◯	◯	◯
Using cognitive challenges to test loudness calibration	◯	◯	◯
Challenging client by moving out of treatment room as appropriate	◯	◯	◯
Being relentless, persistent and redundant in cueing calibration	◯	◯	◯
Recognizing signs of unsuccessful calibration	◯	◯	◯

C5. Hierarchy

	"I've got it" (Well-developed skill)	***"I am getting it"*** (Emerging skill)	***"I don't get it"*** (Need help!)
Using personalized, salient materials; includes varied activities through the week	○	○	○
Providing at least 20-25 minutes of focused, intense hierarchy practice	○	○	○
Varying client tasks when appropriate (e.g. read list in order, then backwards, etc.)	○	○	○
Eliciting 5 minutes of off the cuff speech at the hierarchy level (this may be intermixed throughout the hierarchy)	○	○	○
Using reading stand or holding sheet to promote good head/neck posture	○	○	○
Increasing challenges as client progresses	○	○	○

C6. Homebased practice/carryover

	"I've got it" (Well-developed skill)	***"I am getting it"*** (Emerging skill)	***"I don't get it"*** (Need help!)
Keeping client accountable for home practice each day and offering client opportunities to facilitate compliance with homework, such as Homework Helper or LSVT-C Home Edition	○	○	○
Interactively creating functional, salient carryover exercises with client input	○	○	○
Using feedback about carryover activities to highlight “AH-HA” moments	○	○	○
Organizing information into folder/binder to promote compliance	○	○	○

C7. General

	"I've got it" (Well-developed skill)	***"I am getting it"*** (Emerging skill)	***"I don't get it"*** (Need help!)
Reducing junk minutes	◯	◯	◯
Providing instructions/feedback regarding client's use of good quality, loud voice all the time	◯	◯	◯
Using handouts from LSVT manual as appropriate	◯	◯	◯
Using positive spin as needed	◯	◯	◯
Formulating clear post-treatment recommendations and plan for client	◯	◯	◯

C8. Documentation

	"I've got it" (Well-developed skill)	***"I am getting it"*** (Emerging skill)	***"I don't get it"*** (Need help!)
Recording baseline and final data to demonstrate program outcomes to client	◯	◯	◯
Recording and calculating average SPL, frequency and duration each day of treatment	◯	◯	◯
Tracking client progress in daily/weekly treatment notes	◯	◯	◯
Writing treatment notes that are concise, well-organized and focused on client performance	◯	◯	◯
Writing treatment notes that reflect client progress, challenges and future directions	◯	◯	◯
Writing a progress report that is timely, client-focused and reflective of client progress, functional gains, needs and recommendations	◯	◯	◯

We would appreciate your feedback in regards to the clarity and content of this survey. If you have any comments/feedback please write below:

Thank you very much for completing this questionnaire

If you have any queries please contact:

Patricia Masterson-Algar
School of Healthcare Sciences
Bangor University
Bangor, LL57 2EF
p.m.algar@bangor.ac.uk
01248 383129
