# Supplementary material for: The PD COMM trial: a protocol for the process evaluation of a randomised trial assessing the effectiveness of two types of SLT for people with Parkinson’s disease
Source: Trials. 2017 Aug 29;18:397. doi: 10.1186/s13063-017-2130-1 (PMC5576370; doi:10.1186/s13063-017-2130-1)
Supplement: Supplementary file 2 — PD COMM process evaluation participant interview schedule. (PDF 354 kb) [file 13063_2017_2130_MOESM2_ESM.pdf]

PREAMBLE

Many thanks for taking the time to help with this interview.

This interview is being carried out to gather information about the experiences and opinions of participants in the PD COMM trial. I would like to find out how you feel about having PD and about the treatment you are receiving. I would like to explore how you think the illness affects your body but also your everyday life and in what ways. If at any time you want to stop, or have a break or you don't want to answer a question please let me know. Please be assured that all what you say will remain confidential but may be anonymously quoted.

Can I ask you to confirm that you are happy to continue, and for the conversation to be recorded. If you are not I can make notes instead.

Living with PD

1. **Can you tell me a little bit about when you were first diagnosed? What made you go to the doctor in the first place?**

   - *What sort of problems were you experiencing?*
   - *Did you go to the doctor because a family member or friend recommended doing so?*
   - *Do you normally avoid doctors?*

2. **How much did you know at the time about PD?**

   - *What did the name PD mean to you at that time?*
   - *Where you familiar at all with this illness?*
   - *Are you the sort of person who will find information and will seek information to read (e.g. magazines, the internet)?*

3. **How did you go about seeking help to manage the illness?**

   - *How did you identify what sort of help you were going to need?*
   - *Who were your sources of support and information?*
   - *How do you normally deal with challenges?*

4. **How would you describe yourself as a patient? (Active or passive)**

   - *Do you ask the doctors and therapists all the questions you have? Do you feel comfortable doing that? Do you feel you are in control?*
   - *Do you rather figure things out on your own?*

5. **How do you feel you are coping with the problem you have with your speech?**

    - *Does it worry you?*
    - *Does it get you down?*

6. **What aspects of having PD and problems with your speech do you find more difficult to cope with?**

    - *In terms of your everyday life*
    - *In terms of physical symptoms*
    - *In terms of your mood*

7. **Does having PD and problems with your speech affect your life at home?**

    - *How do you think the people close to you view your illness?*
    - *Do your problems with your speech affect your social and leisure life? In what way?*

Being part of the trial

8. **How have you found being part of the trial?**

    - *How did you find out about it?*
    - *Did your family members encourage you to be part of it? Or health professionals?*
    - *What made you want to join? (e.g. pleasing my family, I wanted to try anything available)*
    - *Was it what you expected? In what way?*

9. **Do you feel therapists and researchers have explained things well?**

    - *Did you have a clear idea of the purpose of the trial?*
    - *Did you know the different groups that you could be randomised to? How did you feel about that? Did it worry you to think you would get put in the 'control group'?*
    - *How did you feel when you were told the group you had been randomized into?*

10. **Has being part of the trial been a big commitment? Was it hard? In what way?**

    - *Has it required you to arrange help from family members (e.g. for lifts)? In what way?*
    - *Has it impacted on your routine? How have you dealt with that?*
    - *How did you deal with the 'homework'? Was it very demanding? In what way?*

11. Have you had second thoughts about joining the trial? Why?

- *Was there times when you thought you shouldn't have joined? Why?*
- *If you could go back in time, would you join the trial again? Why?*

12. How do you feel being part of the trial has changed the way you deal with your illness?

- *Have you learnt more about how to deal with your speech problems and your illness?*
- *Have your family members/friends been happy with your involvement in the trial? Why?*

To end: *I have asked you all the questions I wanted to ask you, is there anything you would like to say or any other issue I haven't mentioned that you would like to discuss?*

Thank you very much for your help.
