# Supplementary material for: The PD COMM trial: a protocol for the process evaluation of a randomised trial assessing the effectiveness of two types of SLT for people with Parkinson’s disease
Source: Trials. 2017 Aug 29;18:397. doi: 10.1186/s13063-017-2130-1 (PMC5576370; doi:10.1186/s13063-017-2130-1)
Supplement: Supplementary file 3 — PD COMM process evaluation therapist interview schedule. (PDF 369 kb) [file 13063_2017_2130_MOESM3_ESM.pdf]

PREAMBLE

Many thanks for taking the time to help with this interview.

This interview is being carried out to gather information from speech and language therapists working as part of the PD COMM trial. Data obtained from the interviews will inform the PD COMM trial's process evaluation. The interview is carried out and recorded with consent from the SLT and transcripts will be made available. Gathered information will remain confidential but may be anonymously quoted.

Can I ask you to confirm that you are happy to proceed, and for the conversation to be recorded?

Icebreaker: **Tell me about your role within the PD COMM trial.**

Are you enjoying it? / Have you enjoyed it?
What intervention have you been delivering (LSVT and/or NHS standard therapy)? Was that very different to what you normally do?

1. **How would you describe speech and language therapy services in your service?**

   - *How do you think what PD COMM proposes fits into the current provision?*
   - *How easy has it been to deliver LSVT? And NHS standard therapy?*
   - *Is LSVT something new to your service?*
   - *Do you think LSVT/NHS standard therapy brings change to your service? In which way?*
   - *How familiar were the people around you to LSVT/NHS standard therapy?*

2. **Tell me a little bit about your previous experience as a SLT and with PD patients**

   - *How has your previous experience impacted on your role in PD COMM?*
   - *Is this the first time you are involved in research?*

3. **How helpful was the guidance you received prior and during the trial regarding how to carry out your role as a PD COMM therapist?**

   - *Did you attend training sessions? Workshops? Webinars? Were they helpful? In which way?*
   - *Was your role well explained to you? Did it make you feel confident about what your role entailed and what you were supposed to do? Why?*

4. **How did the recording documentation methods suit your professional way of working?**

   - *Did you feel that the way you were asked to document and record your work was clear?*
   - *Do you think the documents you have completed clearly reflect the work you've done? Why? Examples?*
   - *Was it what you would have normally done? In what way?*

Delivering the intervention – *the process*

5. **To what extend have you been able to deliver the trial intervention according to plan? Which bits and why?**

    - *How easy or not was it to follow LSVT guidelines? Why?*
    - *How does the LSVT ethos fit with your normal way of working and your personality?*
    - *Was delivering LSVT/NHS standard therapy as straightforward as you thought? Why?*
    - *Do you feel you have been able to tailor your therapy to patients' needs? Why? Was there enough 'flexibility' in the guidelines?*
    - *In what ways have you tailored therapy to patients' needs? Can you give me some examples? Do you think this tailoring is clear in your therapy notes? Why?*

6. **Do you think the way you have delivered the PD COMM interventions (LSVT/NHS standard therapy) has changed with time?**

    - *Do you think you have learned to be 'better at it'? In what way?*
    - *Do you think 'learning' has impacted on how you deliver the interventions? Think about your first patient and your latest patient, can you think of any differences in the way you have approached and worked with them?*

7. **What would you say are the biggest challenges you have faced when delivering the interventions (LSVT/NHS standard therapy)?**

    - *How did you approach the challenges? What did you do to solve problems?*
    - *What sources of support have you reached out to when you have faced a challenge? Trial managers? Personal reflections? Other SLTs?*
    - *Was there enough support available? What support was most useful to you?*
    - *How did you deal with 'unmotivated' patients?*

Delivering the intervention – *the inner setting*

8. **Think about your service/trust. What was it about it that helped or hindered your work? Can you think of specific examples?**

    - *Were your managers on board? In what way?*
    - *What changes did your service have to make, if any, to maximize the success of the trial interventions?*
    - *What changes do you think your service could have made in order to be more receptive of the PD COMM interventions and objectives?*
    - *Do you think the PD COMM purpose and objectives 'fit' with the culture and the mission statement of your service? In what way?*

Intervention impacts

9. Do you believe that the PD COMM interventions (LSVT/NHS Standard therapy) have a positive impact on patients dealing with Parkinson's disease? Why?

- *Do you think that impact has been recorded in the paperwork? How? Why?*
- *How do you think the level of impact has affected your motivations and engagement throughout your involvement in the trial?*
- *Was the impact of the interventions you delivered as you expected? In what way?*

To end: Is there anything else you would like to say or you would like to discuss any further before we finish this interview?
