# Supplementary material for: The PD COMM trial: a protocol for the process evaluation of a randomised trial assessing the effectiveness of two types of SLT for people with Parkinson’s disease
Source: Trials. 2017 Aug 29;18:397. doi: 10.1186/s13063-017-2130-1 (PMC5576370; doi:10.1186/s13063-017-2130-1)
Supplement: Supplementary file 4 — PD COMM process evaluation critical incident report form. (PDF 291 kb) [file 13063_2017_2130_MOESM4_ESM.pdf]

PD COMM THERAPIST CRITICAL INCIDENT ANALYSIS

Therapist:	Participant code:	Date of completion:

Introduction

We are keen to understand the factors that are shaping your work as speech and language therapists and therapy assistants whilst you are working with participants delivering the PD COMM trial interventions. This will help us explain the trial results.

Critical incident technique

We recognise that all of the trial therapists will bring different views, experiences and knowledge to their role and practice. The critical incident technique is a well-established method to systematically analyse the sense that health professionals make of situations that are important to them. Important situations are ones that stand out as being exemplary of good (or poor) clinical experience, or ones that change the way that we think about things. We use a cycle of questions to help us think systematically about a critical incident as follows:

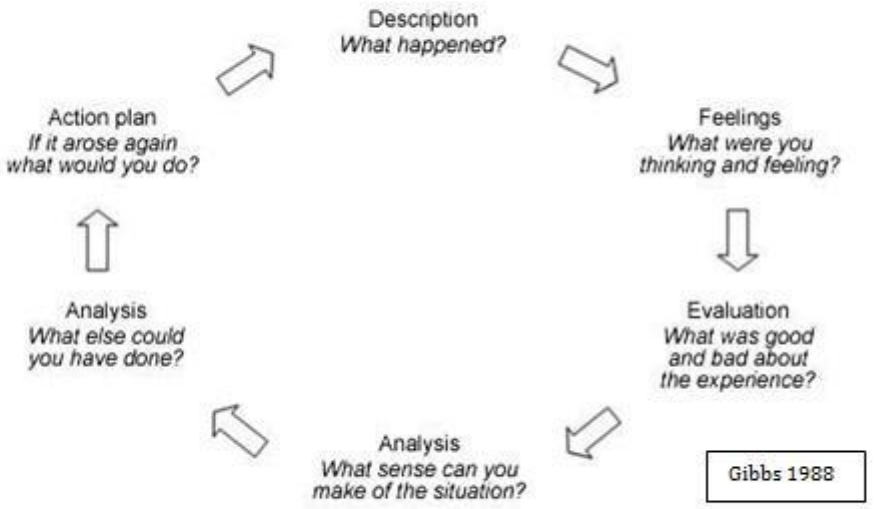

Your role

We would like you to reflect on a number of critical incidents to help us understand your work. Overleaf is a series of questions and prompts to help you do this. There isn't a specific number of critical incidents that you should consider – it's important to focus on incidents that stand out for you. You may find it useful to bring these to clinical supervision and **to include them in your CPD portfolio.**

Please remember confidentiality, and **do not include any identifiable information other than the participant code and date of the critical incident.** Please provide as much detail as you can.

PD COMM THERAPIST CRITICAL INCIDENT ANALYSIS

1. **Choose a critical incident:**
This would be something that stands out for you, such as a successful or unsuccessful encounter with a participant, a particularly successful or unsuccessful treatment, or a specific problem that you have faced in your work in the PD COMM study.

2. **Describe the incident in the box below, including:**
- When and where it happened (time of day, location and social and organisational context)
- What PD COMM intervention were you using and why
- Were you doing anything any differently from what you had been doing for other participants?
- What else happened (who said or did what?)
- What else was going on that influenced what happened

3. **What were your feelings about the critical incident? You might consider:**
- What were you thinking and feeling at the time and just after the incident?
- What were you hoping to achieve
- What led up to the incident?
- How did you deal with the incident?
- How the incident could have been avoided

4. **Evaluation:**

- What was the problem?
- Why was it a problem?
- What would you do if you were the person in the story?
- Who would you ask for help?
- Why does this incident stand out?

5. **Analysis**

- What is going on here – can you explain things that are going on
- Where would you have acted the same as the persons in the story, where would you have acted differently?
- Did a particular mind-set/bias lead to the event

6. **Conclusion**

- Could you have interpreted this event differently from another point of view?
- What can you learn from this episode?

7. **Action plan**

- How could you avoid the problem in the future?
- How could you now solve the problem which already exists?
- How can you prepare yourself to handle such problems?
- What would be your preferred (ideal) option/choice?

Once you have completed your critical incident analysis, please return it by post or email it to the following address:

Patricia Masterson Algar
School of Healthcare Sciences,
Bangor University Fron Heulog
Bangor,
Gwynedd,
LL57 2EF
p.m.algar@bangor.ac.uk

Please keep a copy before you send it both for your CPD portfolio and in case we ask to discuss the critical incident with you.

We may telephone you if we have any questions or points for clarification.

Many thanks for completing this critical incident analysis.
